# Les accidents du traitement anticoagulant dans le Service de Cardiologie du Centre Hospitalier Universitaire Yalgado Ouedraogo de Ouagadougou (Burkina Faso)

**DOI:** 10.11604/pamj.2018.29.135.10650

**Published:** 2018-02-27

**Authors:** Georges Rosario Christian Millogo, Jonas Koudougou Kologo, Georges Kinda, Nobila Valentin Yaméogo, Jean Baptiste Tougma, Yibar Kambiré, Anna Tall Thiam, Benoît Sanon, Jean Yves Toguyeni, André Samadoulougou, Patrice Zabsonré

**Affiliations:** 1 UFR/SDS (Unité de Formation et de Recherche en Science de la Santé), Service de Cardiologie du CHU Yalgado Ouédraogo, Ouagadougou, Burkina Faso; 2 INSSA (Institut National de Science de la Santé), Service de Cardiologie du CHU Yalgado Ouédraogo, Ouagadougou, Burkina Faso; 3 CHU Yalgado Ouedraogo (Centre Hospitalier Universitaire Yalgado Ouedraogo), Service de Cardiologie du CHU Yalgado Ouédraogo, Ouagadougou, Burkina Faso

**Keywords:** Anticoagulant, hémorragie, thrombose veineuse, Anticoagulant, hemorrhage, venous thrombosis

## Abstract

Etudier le profil des patients hospitalisés pour hémorragie sous traitement anticoagulant. Il s’est agi d’une étude rétrospective à visée descriptive sur une période de 02 ans allant du 1^er^ Janvier 2007 au 31 Décembre 2008, dans le Service de Cardiologie du Centre Hospitalier Universitaire-Yalgado Ouédraogo. Tous les patients hospitalisés présentant une hémorragie sous anticoagulant ont été inclus dans l'étude. L’âge moyen des patients était de 49,31 ± 17,68 ans, le sex-ratio était de 2,17. L’infarctus du myocarde était la première indication du traitement anticoagulant avec une fréquence de 21,05%. L’anti Vitamine K (AVK) a été pourvoyeur d’hémorragies dans 63,16% (n = 12) des cas contre 36,84% (n = 7) pour les Héparines de Bas Poids Moléculaire (HBPM); Il s’agissait de 10 cas d’hémorragie majeure et de neuf cas d’hémorragie mineure. La durée moyenne du traitement AVK était de 16 semaines ± 58 semaines. Le saignement par le tractus digestif était le plus fréquent (31,58%) et dans 89,47% des cas, le traitement était associé à un antiagrégant plaquettaire. L’arrêt définitif de l’anticoagulant constituait la prise en charge de l’accident hémorragique dans 73,68%. Il y avait quatre (21,05%) décès. L’inaccessibilité des antidotes comme le sulfate de protamine et les PPSD (Prothrombine Proconvertine Stuart B) constituent un véritable frein à la prise en charge adéquate des complications; mais une meilleure éducation de patients utilisateur de ces drogues constituerait la plus importante des préventions, car plus de 50% de ces accidents sont évitables.

## Abstract (en)

This study aimed to evaluate the profile of patients hospitalized for anticoagulant-induced hemorrhage. We conducted a retrospective, descriptive study within the Department of Cardiology at the Yalgado Ouedraogo Teaching Hospital, in Ouagadougou, over a period of 2 years from 1 January 2007 to 31 December 2008. All hospitalized patients with anticoagulant-induced hemorrhage were included in the study. The average age of patients was 49,31 ± 17,68 years, the sex-ratio was 2,17. Myocardial infarction was the first indication for anticoagulant treatment, with a rate of 21.05%. Anti vitamin K (AVK) was associated with hemorrhage in 63,16% (n=12) of patients versus 36,84% (n=7) of patients treated with low molecular weight heparins (LMWH); 10 patients had major hemorrhage while nine patients had minor hemorrhage. The average duration of Anti vitamin K (AVK) treatment was 16 ± 58 weeks. Hemorrhage in the digestive tract was the most frequent symptom (31,58%) and, in 89,47% of patients, treatment was associated with platelet aggregation. Treatment of hemorrhagic accident was based on definitive cessation of anticoagulant therapy in 73,68% of patients. Four patients (21.05%) died. The inaccessibility to antidotes such as protamine sulphate and PPSD (Prothrombin, Proconvertine, Stuart factor, and anti-haemophilia B factor) constitutes a real obstacle to adequate treatment for complications; a better education of patients receiving these drugs would be the most important preventive measure, because more than 50% of these accidents are preventable.

## Introduction

Les maladies cardiovasculaires sont la première cause de mortalité dans le monde; leur incidence augmente dans tous les pays. Les affections thromboemboliques y occupent une place importante. Les anticoagulants, pierre angulaire dans leur prise en charge, sont aussi responsables de complications non négligeables. Elles sont même la première cause d’hospitalisation pour iatrogénie dans les pays développés [1]. Les anticoagulants sont indiqués dans le traitement curatif et la prévention secondaire de la maladie thromboembolique veineuse et artérielle [1,2]. Pour les indications d’anticoagulation au long cours, ce sont les anti-vitamines K (AVK) qui sont prescrits dans notre contexte, tout comme en France en 2003 [1, 3]. Au Burkina Faso, avec l’allongement de l’espérance de vie [4,5], l’usage des anti thrombotiques connaît un essor par l´augmentation de leurs indications. Ainsi, en l’absence de données sur l’ampleur du problème, il nous a paru opportun de mener cette étude pour évaluer les complications du traitement aux anticoagulants et leur prise en charge dans le service de cardiologie du Centre Hospitalier Universitaire Yalgado Ouédraogo.

## Méthodes

Nous avons entrepris, dans le service de cardiologie du CHU Yalgado Ouédraogo de Ouagadougou, durant la période allant du 1^er^ Janvier 2007 au 31 Décembre 2008, une étude rétrospective à visée descriptive. Les patients inclus, étaient ceux traités par anticoagulants (AVK et/ou héparine) et hospitalisés pour hémorragie et les patients ayant présenté une hémorragie sous anticoagulants pendant leur hospitalisation. Pour chaque patient, une fiche de collecte de données a été utilisée. Elle comportait, les données anthropométriques, les antécédents, l’indication et les doses de traitement anticoagulant, l’ancienneté de sa prescription et les traitements associés. Les paramètres biologiques dosés étaient essentiellement, l’INR (avant et au moment de l’accident), le taux d’hémoglobine et de plaquettes, la créatininémie. Les autres renseignements étaient la localisation et l’importance du saignement, le traitement entrepris (arrêt de l’anticoagulant, prescription d’antidote, transfusion sanguine ou remplissage vasculaire par des macromolécules ou du plasma) et l’évolution du patient en cours d’hospitalisation. Les données étaient saisies et analysées à l’aide du logiciel Epi Info version 3.5.1. Etait définie comme hémorragie majeure toute hémorragie fatale, intracrânienne, rétro péritonéale, ou ayant nécessité une transfusion sanguine. Le surdosage aux AVK regroupait toutes les situations où l’INR était > 6 en l’absence de toute manifestation hémorragique.

## Résultats

**Figure 1:**
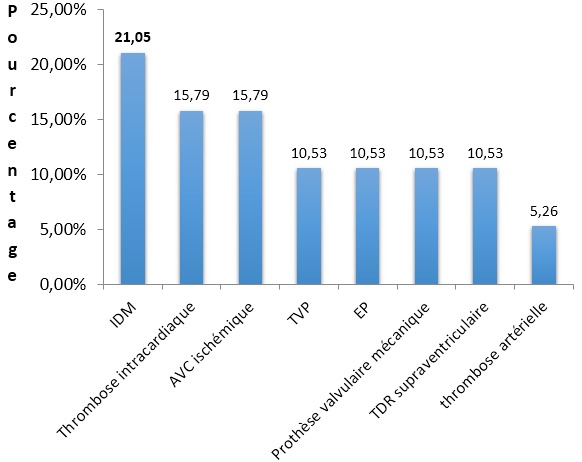
Indications du traitement anticoagulant. (IDM: infartus du myocarde, AVC ischémique: accident vasculaire cérébraux ischémique, TVP: thrombose veineuse profonde, TDR: trouble du risque, EP: embolie pulmonaire)

Dix-neuf (19) des 230 patients sous anticoagulant durant la période d´étude avaient présenté une complication aux anticoagulants (8,26%). L’âge moyen de nos patients était de 49,31 ± 17,68 ans avec des extrêmes de 19 et 78 ans et 78,95% avaient moins de 65 ans. Il y'avait 13 hommes pour 6 femmes, soit un sex-ratio de 2,17. Les femmes au foyer étaient les plus concernées avec une fréquence de 31,58% (n = 6); 78,94% de nos patients (n = 15) résidaient dans la ville de Ouagadougou, trois (03) en zone rurale et un patient résidait dans une autre ville. Aucun patient n’avait un antécédent d’hémorragie aux anticoagulants. Le principal antécédent retrouvé en plus de la pathologie à l’origine de l’indication de l’anticoagulation chez nos patients était l’HTA dans 36,84% (n = 7) des cas, l’intoxication alcoolique 26,32% (n = 5), le tabagisme 15,79% (n = 3), la contraception hormonale et le diabète 10,53% (n = 2). Dix-sept (17) patients (89,47%) avaient un traitement antiagrégant plaquettaire en association avec le traitement anticoagulant au moment de l’accident. L’IDM était le principal motif du traitement anticoagulant avec une fréquence de 21,05%, suivi de la thrombose intracardiaque dans 15,79% des cas, la Figure 1 montre la répartition des patients victimes d’accident hémorragique selon l’indication d’anticoagulation. L’AVK était le plus grand pourvoyeur d’hémorragies, 63,16% (n =12) cas contre 36,84% (n = 7) pour les héparines de bas poids moléculaire (HBPM). Il s’agissait de 10 cas d’hémorragie majeure et de neuf cas d’hémorragie mineure. La quasi-totalité des patients prenaient la même molécule autant pour l’AVK que pour l’héparine de bas poids moléculaire, en effet, 96,17% des patients étaient sous énoxaparine sodique et 96,03% sous fluindione. La durée moyenne du traitement AVK était de 16 semaines ± 58 semaines. Un ou plusieurs facteurs favorisant l’accident hémorragique ont été retrouvés chez nos patients, le Tableau 1indique la répartition des associations morbides rencontrées chez les patients victimes d’accident. Le facteur la plus retrouvé était l’association médicamenteuse, notamment les antiagrégants plaquettaires dans 89,47% des cas d’accident hémorragique, l’HTA dans 36,84% des cas et les lésions digestives dans 31,58% des cas. Les hémorragies du tractus digestif étaient l’accident le plus retrouvé dans 6 cas (31,58%) suivies des gingivorragies avec 5 cas (26,32%) (Tableau 2). L’INR (International Normalized Ratio) moyen chez nos patients au moment de l’accident était à 11. Face aux cas d’hémorragie majeure, la conduite à tenir a été, l’arrêt définitif du traitement anticoagulant en cours dans sept (07) cas, l’arrêt temporaire avec des contrôles rapprochés de l’INR dans trois cas, et reprise du traitement AVK à distance; cinq (05) patients de ce groupe ont bénéficié d’une transfusion sanguine et seulement trois (03) patients se sont vus administrer de la vitamine K, deux par voie orale et un par voie parentérale. Dans le groupe des hémorragies mineures, l’attitude thérapeutique a consisté à l’arrêt définitif du traitement AVK dans sept (07) cas avec administration de vitamine K chez un patient, les deux autres patients ont bénéficié d’un arrêt temporaire du traitement anticoagulant. Il y avait quatre (04) décès, soit une mortalité de 21%.

**Tableau 1:** Répartition des associations morbides rencontrées chez les patients ayant présenté une complication hémorragique dans le service de cardiologie du CHU Yalgado, Ouagadougou, Burkina Faso

Association morbide	Effectif	Pourcentage (%)
Polythérapie	17	89,47
Hypertension artérielle	7	36,84
Lésion digestive	6	31,58
Insuffisance rénale	4	21,05
Insuffisance hépatique	4	21,05
Obésité	3	15,79
Diabète	2	10,53
Fibrome utérin	1	5,26
Artériopathie	1	5,26

**Tableau 2:** Répartition des complications hémorragiques selon leur siège

Siège de l'hémorragie	Effectif	Pourcentage (%)
Hémorragie digestive	6	31,58
Hémoptysie	5	26,32
Gingivorragie	4	21,05
Hémorragie Intra Crânienne	3	15,79
Hématome	3	15,79
Métrorragie	2	10,53
Hématurie	1	5,26
Epistaxis	1	5,26

## Discussion

Nous avons trouvé dans notre étude une incidence de 8,26% d’accident aux anticoagulants dont 63,16% liées aux AVK contre 36,84% aux HBPM. F.D. KAMDEM en République de Côte d’Ivoire (RCI) retrouvait en 2003, 28,8% de cas d’accidents hémorragiques dont 5,8% majeurs [2] alors que P. POUYANNE en France, trouvait 13% d’admission pour accidents aux anti-thrombotiques [6]. Y. BENHAMOU en 2009 dans une étude de cohorte regroupant 530 patients suivis dans les centre et de suivi et de conseil des traitements anticoagulants (CSCTA) en France, retrouvait 4,9 pour 100 patients-année de cas d’accidents aux anti-thrombotiques [7]. Quant à M. PIRMOHAMED, il retrouvait dans une étude prospective incluant 18820 patients portant sur les hospitalisations pour effets indésirables des médicaments en Grande Bretagne 10,5% d’admissions pour hémorragies sous AVK (warfarine) [8]. Cette faible proportion de cas d’accidents hémorragiques de notre étude comparativement aux autres pourrait s’expliquer par une différence de critères d’inclusion. En effet, dans notre étude (contrairement aux autres), seuls les patients chez qui le traitement anti-thrombotique était accessible ont été mis sous ce traitement. Cette hypothèse est d’autant plus plausible que la présence de centres spécialisés dans la gestion de l’anticoagulation réduit de façon significative les complications du traitement AVK comme l’a montré l’étude sur les cliniques d’anticoagulants (CAC) menée par P. LEGER et al. [1]. P. POUYANNE [6] retrouvait un âge significativement plus élevé des patients hospitalisés pour effets indésirable de médicaments comparativement aux hospitalisations pour autre motif (60,5 contre 52,9; p = 0,009) avec une susceptibilité féminine significativement plus élevée et une corrélation avec l’âge. Notre étude tout comme celle de POUYANNE a trouvé un âge moyen des patients ayant présenté un accident hémorragique inférieur à l’âge de référence des scores HAS-BLED et BRI qui est de 65 ans [9]. Cette relative jeunesse de la population pourrait s’expliquer dans notre étude par la présence de sujet très jeunes (19 ans) et dans les deux études par le fait qu’un seul item sur sept des critères d’évaluation du risque hémorragique a été considéré, les autres n’ayant pas été évalués. La prédominance masculine dans notre étude pourrait s’expliquer par le « visage féminin » de la pauvreté et l’absence d’une sécurité sociale prenant en charge les soins de santé dans notre pays. Le traitement anti-thrombotique serait plus accessible aux hommes qu´aux femmes [10,11].

Les indications les plus fréquentes du traitement anti-thrombotique chez les patients qui ont présenté un accident hémorragique dans notre études étaient représentées par l’infarctus du myocarde (IDM) dans 21,05% et la thrombose intracardiaque 15,79% des cas. KAMDEM en Côte d’Ivoire et BEHAMOU en France, retrouvaient une prévalence des troubles du rythme cardiaque comme première indication au traitement anti-thrombotique dans 19,5% et 55% des cas respectivement [2,7]. Cette différence de résultats entre nos résultats et ceux de la littérature pourrait être dû au fait que tous les patients éligibles n’avaient pas bénéficié du traitement anti-thrombotique du fait de son inaccessibilité financière. L’AVK a été le plus gros pourvoyeur d’hémorragies dans notre étude et ce résultat est concordant avec ceux des études de POUYANNE [6] et les conclusions de PAUTAS sur la gestion des accidents aux anticoagulants aux urgences [3] qui placent les AVK au premier rang des anti-thrombotiques pourvoyeurs d’accident hémorragiques. De même, ces études retrouvent une fréquence plus élevée des accidents mineurs. En ce qui concerne l’HTA plusieurs études [2, 12, 13] ont montré que l’HTA même traitée est associée à une augmentation du risque hémorragique au cours du traitement anticoagulant. Dans notre étude, l’hypertension artérielle était le principal facteur de risque cardiovasculaire associé et retrouvé dans 36,84% des cas sans qu’on ait pu démontrer un lien de causalité. Selon plusieurs études [12-15], la co-prescription d’un médicament potentialisant l’effet des AVK est un facteur prédictif de saignement. Dans notre étude, les AINS et les antibiotiques sont retrouvés dans la majorité des cas.

Dans notre étude, le siège le plus fréquent des accidents hémorragiques était le tractus digestif avec 31,58% des cas. POUYANNE et PAUTAS [3,6], retrouvaient également une prédominance digestive dans la localisation des accidents hémorragiques. L’INR médian dans notre étude était comparable à celui retrouvé dans la série de Ben Ameur [16]. La prise en charge de ces complications hémorragiques a fait l’objet de récentes recommandations en Avril 2008. La base du traitement médicamenteux est la Vitamine K parfois associées aux PPSB (Prothrombine Proconvertine Stuart B), et peut être complétée par un geste d’hémostase (artériographie avec embolisation, chirurgie d’hémostase). Les recommandations de l’HAS en avril 2008 portaient sur trois situations majeures: les surdosages asymptomatiques, la survenue d’une hémorragie, spontanée ou traumatique, associée ou non à un surdosage et la prise en charge lors d’une chirurgie ou d’un acte invasif [17,18]. Notre attitude face à ces accidents a été essentiellement l’arrêt de l’anticoagulation chez tous les patients, en revanche la vitamine K a été très peu utilisée et cette attitude est retrouvée dans plusieurs séries [16, 17, 19, 20] où la vitamine K était utilisée dans moins de 20% des cas. Dans notre service où le PPSB n´est pas disponible, la prise en charge des hémorragies graves est peu efficiente.

Les accidents hémorragiques aux anticoagulants sont responsables d’une forte morbi-mortatilté. Dans notre série, il y avait un fort taux de mortalité mais il était inférieur à celui de Serghini au Maroc qui trouvait 40% de mortalité dans un service réanimation [17]. Le taux de mortalité dans notre étude était par contre plus élevé que celui de la série de SIGURET et al.l qui a enregistré trois cas de décès sur les 59 cas de surdosage [21]. Il faut noter que tous les auteurs s’accordent sur la nécessité de la mise en place de clinique d’anticoagulant, véritable interface entre les médecins et les patients dont le rôle est de sensibiliser et d’éduquer ces derniers dans les premiers instants de la mise en route du traitement anticoagulant [18, 22-24].

## Conclusion

Le traitement anti-thrombotique malgré son accessibilité limitée connaît une utilisation importante dans notre service où les complications liées à son utilisation sont aussi réelles. L’âge avancé et la polymédication sont des facteurs de risque de saignement. Le mésusage de ces anticoagulants est responsable d’une mortalité élevée. Autant l’accessibilité du traitement est limitée, autant celle des antidotes comme le sulfate de protamine et les PPSD constituent un véritable frein à la prise en charge adéquate des complications. D’où, la nécessité d’une prescription raisonnée prenant en compte toutes les comorbidités visant un rapport bénéfice-risque satisfaisant.

### Etat des connaissances actuelles sur le sujet

- C’est la première cause d’hospitalisation pour iatrogénie dans les services de cardiologie dans les pays développés;
- Les anticoagulants sont la pierre angulaire du traitement de maladies thromboemboliques et des cardiopathies emboligènes.

### Contribution de notre étude à la connaissance

- L’émergence de ces cardiopathies emboligènes dans nos pays en dévéloppements;
- L’insuffisance dans la prise en charge des complications liées à l’utilisation des anticoagulants;
- Le manque de moyen pour acquérir et utiliser les anticoagulants oraux directs plus faciles à manipuler.

## Conflits d’intérêts

Les auteurs ne déclarent aucun conflit d'intérêts.

## Remerciements

Les auteurs remercient Dr Kisito Nagalo pour sa précieuse contribution à la rédaction du protocole d'étude.

## References

[1] Leger P, Cambus JP, Boneu B, Boccalon H (2003). Les cliniques d’anticoagulants. Sang Thromb Vaiss..

[2] Kamdem FD, Adoubi AK, Diby FK, Harding D, Ekra A (2007). Utilisation des antithrombotiques en Afrique Subsaharienne: expérience de l’Institut de cardiologie d’Abidjan. Médecine d'Afrique Noire..

[3] Pautas E, Mitha N, Monti A, Boddaert J, Gouin-Thibault I (2011). Gestion des accidents des anticoagulants aux urgences. URGENCES..

[4] The World BANK Perspective Monde, Espérance de vie au Burkina Faso.

[5] INSD Évolution de l’espérance de vie à la naissance, Tableau 03.01..

[6] Pouyanne P, Haramburu F, Imbs JL, Bégaud B (2000). Admissions to hospital caused by adverse drug reactions: cross sectional incidence study. BMJ..

[7] Benhamou Y, Le Cam-Duchez V, Schneller JM (2009). Expérience d’un centre de suivi et de conseil des traitements anticoagulants oraux en médecine de ville: résultats à cinq ans. Rev Med Interne..

[8] Pirmohamed M, James S, Meakin S, Green C (2004). Adverse drug reactions as cause of admission to hospital: prospective analysis of 18 820 patients. BMJ..

[9] Wester JP, De Valk HW, Nieuwenhuis HK, Brouwer CB, Van der Graaf Y (1996). Risk factors for bleeding during treatment of acute venous thromboembolis. Thromb Haemost..

[10] PNUD Ministère de l’habitat et de l’urbanisme Burkina Faso. Stratégie de lutte contre la pauvreté urbaine..

[11] Arnsten JH, Gelfand JM, Singer DE (1997). Determinants of compliance with anticoagulation: a case control study. Am J Med..

[12] Casais P, Sánchez Luceros A, Meschengieser S, Fondevila C, Santarelli M, Lazzari M (2000). Bleeding risk factors in chronic oral anticoagulation with acenocumarol. Am J Hematol..

[13] Landefeld CS, Byeth RJ (1993). Anticoagulant related bleeding: clinical, epidemiology, prediction and prevention. Am J Med..

[14] Landefeld CS, Goldman L (1989). Major bleeding in outpatients treated with warfarIn: incidence and prediction by factors known at the start of outpatient therapy. Am J Med..

[15] Palareti G, Leali N, Coccheri S (1996). Bleeding complications of oral anticoagulant treatment: an inception-cohort, prospective collaborative study (ISCOAT). Lancet..

[16] Ben Ameur Y, Chaabane O, Zairi I, Longo S, Battikh K, Slimane ML (2009). Les accidents hémorragiques graves sous AVK: études descriptive et pronostic. Tunisie Médicale..

[17] Serghini I, Aissaoui Y, Quamouss Y, Sedikki R, Taj N, Alaoui JS, Zoubir M, Boughanem M (2012). Les accidents aux AVK: étude rétrospective à propos de 30 cas. Pan Afr Med J..

[18] Boccalon H (2006). La clinique des anticoagulants: un concept incontournable. Ann Cardiol Angeiol (Paris)..

[19] Fihn SD, Mc Donell M, Martin D (1993). Risk factors for complications of chronic anticoagulation: a multicenter study. Ann Intern Med..

[20] Constans J, Sampoux F, Jarnier P (1999). Complications hémorragiques des anti vitaminiques K: à propos de 75 patients hospitalisés. J Mal Vasc..

[21] Siguret V, Esquirol C, Debray M, Gouin I, Andreux JP, Pautas E (2003). Surdosage en antivitamine K dans une population de patients hospitalisés âgés de plus de 70 ans. Presse Med..

[22] Lacroix P, Portefaix O, Boucher M (1994). Conditions de survenue des accidents hémorragiques intracrâniens des AVK. Arch Mal Coeur Vaiss..

[23] Launbjerg J, Egeblad H, Heaf J (1991). Bleeding complications to oral anticoagulant therapy: multivariate analysis of 1010 treatment years in 551 outpatients. J Intern Med..

[24] Bounameaux H (1994). Complications hémorragiques des anticoagulants en angiologie. J Mal Vasc..

